# Bare metal stent application to prevent limb occlusion in iliac arteries with severe tortuosity during an endovascular aneurysm repair: a cohort study

**DOI:** 10.3389/fcvm.2024.1401929

**Published:** 2024-12-04

**Authors:** Xiong Zeng, Zhinan Ju, Xixi Min, Xiande Zeng, Wei Chen, Kanghui Dai, Weimin Zhou, Jiehua Qiu

**Affiliations:** ^1^ Department of Vascular Surgery, The Second Affiliated Hospital of Nanchang University, Nanchang, Jiangxi, China; ^2^ Department of Vascular Surgery, Jiangxi Provincial People’s Hospital, The First Affiliated Hospital of Nanchang Medical College, Nanchang, Jiangxi, China

**Keywords:** abdominal aortic aneurysm, limb graft occlusion, endovascular aortic aneurysm repair, bare metal stent, interventional therapy

## Abstract

**Background:**

The risk of limb graft occlusion (LGO) after endovascular aneurysm repair (EVAR) is increased by severe tortuosity of the iliac artery. A bare metal stent (BMS) may protect LGO, according to the hypothesis of this single-center retrospective analysis.

**Methods:**

All patients undergoing elective EVAR with a bifurcated stent graft between January 2012 and June 2022 were included in this cohort study. Patients demonstrating significant tortuosity at the iliac angle were incorporated into this study and classified into two groups: group A comprised those who received a BMS. In contrast, group B consisted of those who did not receive a BMS. The primary outcomes were the incidence of limb occlusion and technical success during the follow-up period. However, secondary outcomes included perioperative mortality, external iliac angioplasty, and crossed-limb techniques.

**Statement:**

This study has been reported as being in line with the STROCSS criteria.

**Result:**

A total of 157 patients (mean age = 71.6 ± 8 years) with infrarenal abdominal aortic aneurysms were enrolled. In total, 50 individuals were included in group A, while 107 were in group B. Overall technical success was 100%, and only one (2%) patient from group A and 17 (15%) from group B suffered from limb occlusion during follow-up (*p* < 0.05). At a mean follow-up imaging duration of 28.7 ± 23.6 months (range 1–124), the estimated primary limb patency at 2 years was 98% for the BMS group and 84% for the non-BMS group (*p* < 0.05). There were no changes in perioperative mortality or crossed-limb procedures between the BMS and non-BMS groups. Nonetheless, there were disparities in external iliac angioplasty between the two groups during the follow-up period (*p* < 0.05).

**Conclusion:**

Deploying a BMS inside the iliac artery to prevent or treat limb occlusion is a safe and effective strategy, with clear prolonged outcomes concerning patency and re-interventions.

## Introduction

Endovascular aneurysm repair (EVAR) has been evolving steadily since Parodi's groundbreaking endovascular surgery on an infrarenal abdominal aorta aneurysm (IAAA) in 1991. The availability of endovascular treatment options has increased recently as a result of scientific and technological developments. However, the complications, including endo-leak and limb occlusion (1), have also increased. Limb graft occlusion (LGO) (2) is a severe complication after endovascular repair of an abdominal aorta aneurysm (AAA) and occlusion rates ranging from 2.7% to 10.6% have been reported (3, 4). LGO usually occurs 6 months after an EVAR (5). The common iliac artery (CIA) is a conduit that supplies blood flow to the internal iliac and nourishes the distal extremities. Acute limb ischemia, rest discomfort, claudication, and lower limb necrosis could arise from an occlusion of this artery. Kinking or excessive stent graft oversizing, extension down to the external iliac artery (EIA), and calcification and extreme angulation of the IAs are some risk factors linked to limb occlusion. After an EVAR, severe tortuosity is the primary occlusive factor (6–9). Bare metal stent (BMS) technology has been proposed to prevent LGO (10, 11).

This investigation aimed to elucidate the outcome of BMS employed inside a tortuous IA to prevent limb occlusion after an EVAR.

### Patients and methods

Study design: This retrospective cohort study was conducted at a single center, involving patients who received elective endovascular aneurysm repair for abdominal aortic aneurysms from January 2012 to June 2022. Clinical and anatomical data and operative care outcomes were collected from these patients. The ethics committee at the author's hospital approved the protocol.

Inclusion criteria: (1) Patients who underwent an EVAR with an IAAA, (2) patients with complete clinical data and surgical information, and (3) patients with severe tortuosity of the IA.

Exclusion criteria: (1) Patients with no severe tortuosity of the IA, (2) patients with pseudo-aneurysm of the abdominal aorta, (3) patients with a thoracoabdominal aortic aneurysm, (4) patients with complicated aortic dissection, (5) patients with non-“Y” stent surgery, (6) patients with hybrid or compound surgery, (7) patients with incomplete data, (8) and patients with a ruptured AAA.

Clinical data included ages, diseases, and gender. Pre-operative anatomical data from computed tomography angiography (CTA) were assessed, which included the diameter of the CIA, EIA, and AAA. Severe tortuosity was defined as an acute angle (>60°) of the pathway of the CIA or when IA was observed as visually doubled or more on an axial CTA slice (12). Two cohorts of patients with severe tortuosity of the iliac artery were identified: group A included patients who had a BMS inserted into an abdominal aortic endograft during an EVAR. The auto vessel's diameter, as measured by the pre-operative CTA, defined the size or diameter. The length of the twisted angle determined the length of the BMS (BARD, E-Luminexx™, USA), and fixation needed an anchoring area of at least 3 cm. The length of the BMS was measured with a gold-labeled catheter through a long guide wire, not a stiff wire. Group B comprised patients who did not undergo BMS treatment. The diameter was measured from the outer wall to the outer wall, perpendicular to the long axis of the vessels. When over 50% of the vascular circumference exhibited thrombi or calcification, it was designated a mural thrombus and calcification (13). Balloon dilation was performed according to the anatomical diameter before graft stent placement, and a BMS was to provide radial support for patients with these kinds of vascular stenoses. Stent graft properties include extending to the EIA, regardless of whether a BMS is planted. Physical examinations and CTA scans were followed up after 1, 6, and 12 months and once a year. When the clinical signs indicated LGO, CTA was performed.

### Statistics

SPSS (26.0 version) was utilized for data assessment, and count data were determined by the chi-square test and expressed by percentage (%) and the number of cases (n). Normally distributed data were compared and assessed by a *t*-test and depicted as mean ± standard deviation (x ± s). For the measurement of data with a non-positive distribution, the median (interquartile range) was employed, followed by the sum test. A univariate logistic regression analysis determined the study variables’ crude odds ratios (ORs) and 95% confidence intervals (95% CIs). Furthermore, study variables with statistical significance were included in a multivariate logistic regression analysis. Follow-up outcomes, including the incidence of limb occlusion, were subjected to Kaplan–Meier's life table analysis. All the statistical measurements were two-sided, and a *p*-value < 0.05 was termed statistically significant.

## Results

Over the 10-year study period, 157 individuals were included based on specified inclusion and exclusion criteria, comprising 144 (91.7%) men and 13 (8.3%) women, with a mean age of 71.6 ± 8.6 years. Group A comprised 50 patients, 46 men (92%) and 4 women (8%), with a mean age of 71.8 ± 6.5 years. The cohort exhibited a history of hypertension in 32 patients (64%), diabetes in 2 patients (4%), renal insufficiency in 2 patients (4%), thrombus in 42 patients (84%), and calcification in 6 patients (12%). In group B (107 patients), there were 98 (92%) men and 9 (8%) women (mean age = 71.5 ± 7.1 years). These patients had been previously diagnosed with hypertension [57 patients (54%)], diabetes [7 patients (7%)], renal insufficiency [9 patients (10%)], thrombus [78 patients (73%)], and calcification [14 patients (13%)]. Table 1 presents each group's baseline manifestations, comorbidities, clinical symptoms, IA tortuosity, and procedure-related data. No significant difference was observed between the cohorts in the routine blood test and comorbidities (Table 1).

**Table 1:** Clinical characteristics of patients with or without bare metal stents.

Variable	BMS (*n* = 50)	Non-BMS (*n* = 107)	*p*-value
Age (years)	71.88 ± 6.51	71.53 ± 7.1	0.88
Sex (male)	46 (92%)	98 (92%)	—
Hypertension	32 (64%)	57 (54%)	0.22
Diabetes	2 (4%)	7 (7%)	0.45
Renal insufficiency	2 (4%)	9 (10%)	0.34
Thrombus	42 (84%)	78 (73%)	0.12
Calcification	6 (12%)	14 (13%)	0.50
Thrombocyte	165.4 ± 69.4	168.7 ± 64.4	0.49
Hemoglobin	119.8 ± 18.33	117.23 ± 20.9	0.69
Hematocrit	3.95 ± 0.7	3.87 ± 0.74	0.58
Hemameba	7.98 ± 3.38	8.43 ± 3.3	0.26
AAA diameter (cm)	5.51 ± 1.40	5.80 ± 1.41	0.05
CIA diameter (left) (cm)	2.25 ± 1.40	1.84 ± 0.9	0.06
CIA diameter (right) (cm)	2.1 ± 1.1	1.9 ± 0.98	0.32
EIA diameter (left) (cm)	0.93 ± 0.15	0.93 ± 0.20	0.61
EIA diameter (right) (cm)	0.92 ± 0.14	0.99 ± 0.49	0.94

AAA, abdominal aorta aneurysm; CIA, commoniliacartery; EIA, externaliliacartery.

**Figure 1:**
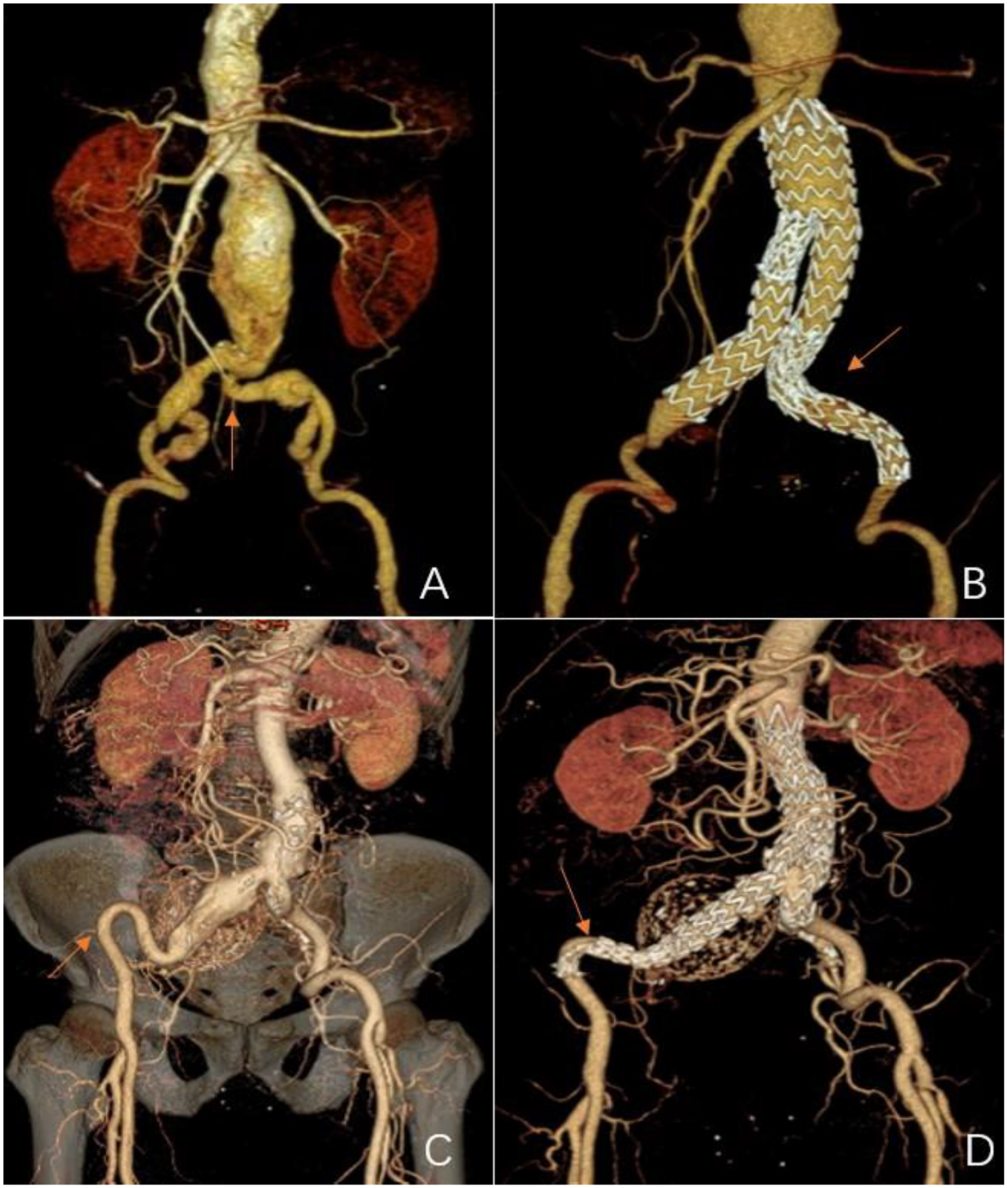
Representations of metal bare stents placed in the common or external iliac arteries. **(A)** and **(C)** Pre-operative 3D CT scans showing severe tortuosity of the left CIA (red arrow), and the double iliac sign of the right EIA (red arrow). **(B)** A post-operative 3D CT scan 18 months after EVAR of a bare metal stent that was placed in the left CIA (red arrow,) showing stent patency. **(D)** A post-operative 3D CT scan 124 months after an EVAR of the other bare metal stent that was placed in the right EIA (red arrow) showing stent patency.

**Figure 2:**
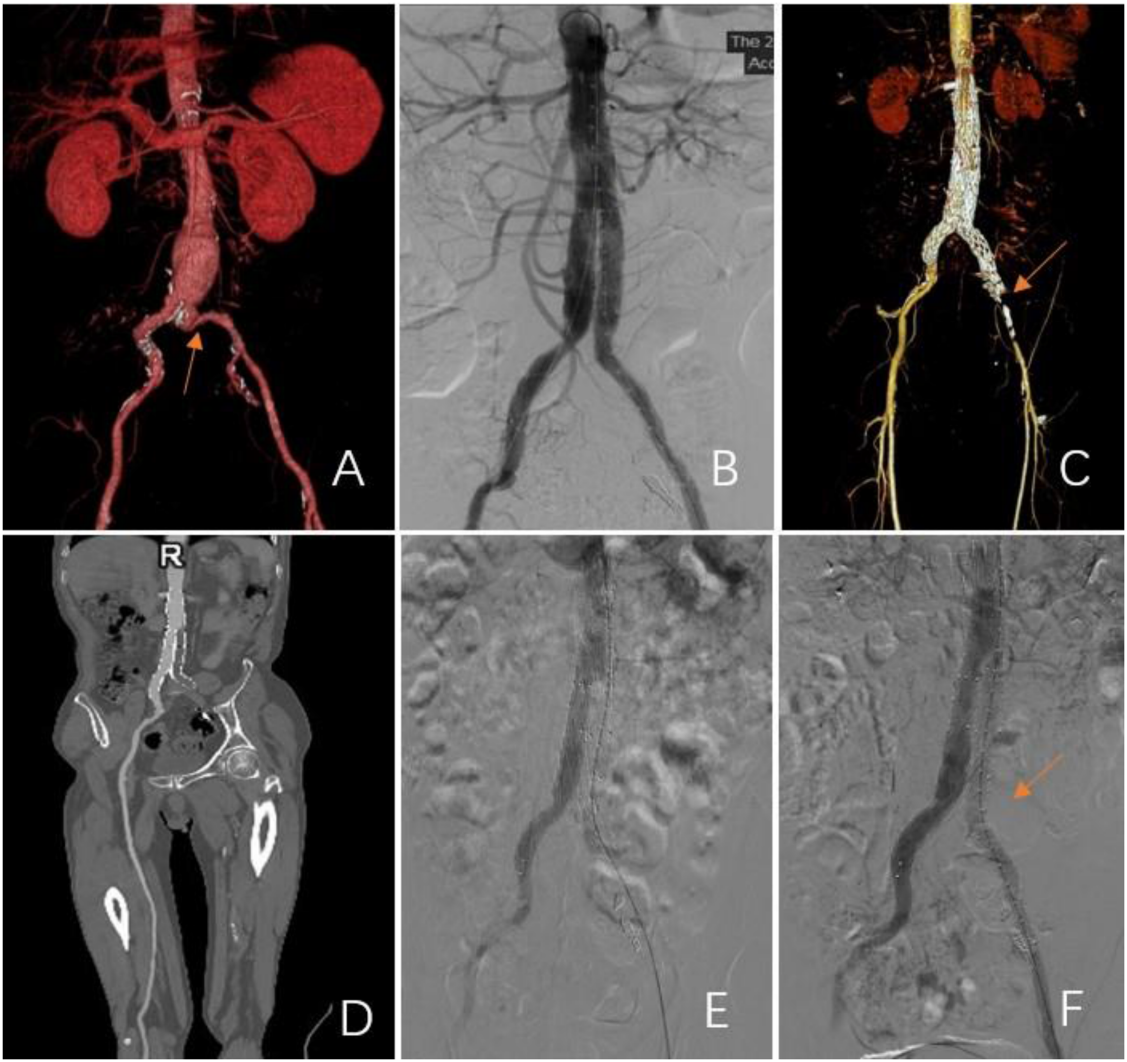
Representations of limb graft occlusion in the group without a BMS. **(A)** A pre-operative 3D CT scan showing severe tortuosity of the iliac artery in the left limb (CIA; red arrow). **(B)** Angiogram showed stent patency after an EVAR. **(C–E)** At 3 months post-procedure, the left limb was occluded. Subsequent 3D CT scans and angiograms confirmed occlusion of the left common iliac artery. **(F)** A bare metal stent was inserted to maintain patency.

**Figure 3:**
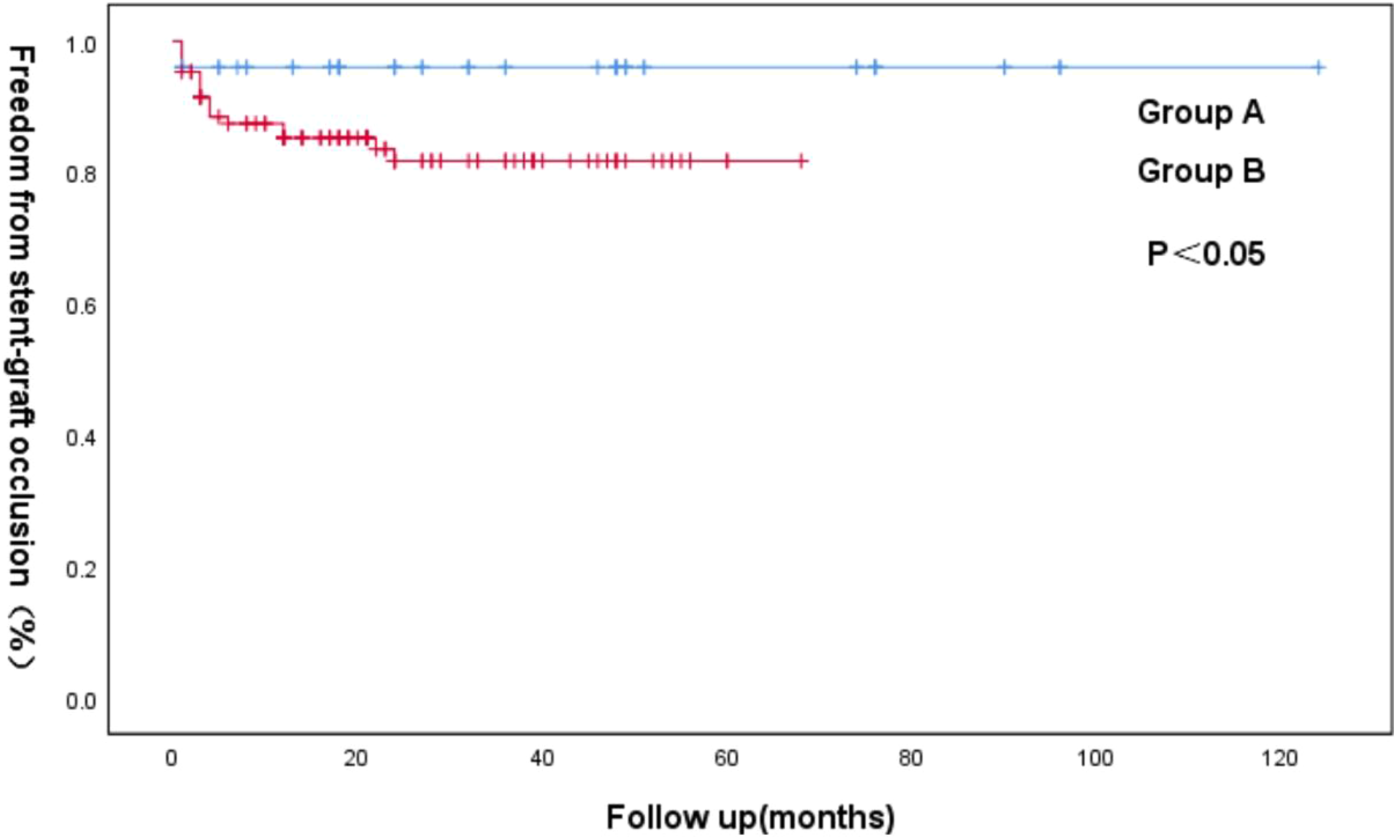
Kaplan–Meier curve of freedom from iliac limb occlusion for the groups A and B following an EVAR.

According to a pre-operative CTA, 157 patients suffered from severe tortuosity in the CIA or the EIA, and 50 of them were implanted with a BMS (Figure 1). Table 2 contains BMS-related data. With 66% vs. 44%, *p* = 0.05, the stent insertion rate in the EIA was higher in the BMS individuals than in the non-BMS individuals. In the BMS group, crossed-limb procedures accounted for 8%; in the non-BMS cohort, they accounted for 12% (*p* ≤ 0.05). The technical success rates for both cohorts were 100% (Table 3). The LGO was 2.0% (1/50) in the BMS cohort and 15% (17/107) in the non-BMS cohort (*p* < 0.05) (Table 3, Figure 2). Most limb occlusions transpired 6 months post-operation (Figure 3), with an overall LGO rate of 11.5% (18/157) in the entire cohort.

**Table 2:** BMS characteristics: type (all BMS are BARD), diameter, and lengths.

BMS characteristics	*N* = 50	%
diameter/lengths:10 mm/80 mm	13	26%
12 mm/80 mm	4	8%
14 mm/80 mm	2	4%
10 mm/100 mm	13	26%
12 mm/100 mm	7	14%
14 mm/100 mm	2	4%
10 mm/120 mm	3	6%
14 mm/120 mm	4	8%
8 mm/60 mm	2	4%
Placement in left	23	46%
Cross the hypogastric artery	33	66%

**Table 3:** Comparison of outcomes between the BMS and non-BMS group.

Outcome	BMS (*n* = 50)	Non-BMS (*n* = 107)	*p*-value
Technical success	100%	100%	—
Limb occlusion	2% (1/50)	15%( 17/107)	0.01
External iliac angioplasty	66% (33/50)	44% (48/107)	0.01
Crossed-limb techniques	8% (4/50)	12% (13/107)	0.4
Perioperative mortality	0% (0/50)	1.8% (1/107)	—
Endograft device type			
Medtronic (Endurent)	29 (58%)	48 (45%)	
Percutek	0 (0%)	10 (9%)	
Cook (Zenith)	0 (0%)	3 (3%)	
MicroPort (Minos/Aegis)	11 (22%)	22 (21%)	
LifeTech (Ankura)	10 (20%)	24 (22%)	

There were differences in the clinical presentations. In group A, only one LGO was observed. The patient had acute limb ischemia and was treated with catheter-directed thrombolysis immediately. In group B, two patients experienced abdominal pain, eight patients experienced claudication, and seven patients experienced acute limb ischemia of grades 2a (*n* = 5) and 2b (*n* = 2). Seven patients received prompt medical attention. Four individuals (24%) received a successful Fogarty catheter embolectomy (FCE) followed by balloon dilatation (one case). Nine patients had a BMS implanted (51%) after either a thrombectomy, catheter-directed thrombolysis, or AngioJet (Boston Scientific) mechanical thrombectomy. Two patients (12.5%) chose anatomic bypasses and FCE and another two individuals received conservative management (12.5%). They were observed to have unilateral intermittent claudication (distance > 500 m) 4 or 18 months after the surgery and no cold feet.

Table 4 displays the outcomes of the LGO univariate analysis. The risk of LGO was substantially correlated with five variables: graft kinking, arteriosclerotic obliterans (ASO), calcification, antiplatelet treatment, and sex. Calcification and graft kinking were also crucial in the multivariate logistic regression (Table 5).

**Table 4:** Univariate logistic regression derived ORs and 95% CIs for LGO, controlling for study covariates.

Variable	Category or increment	Univariate logistic regression analysis OR (95%CI)	*p*-value
Sex (male)	Yes or no	4.476 (1.256–14.515)	0.014
Smoking	Yes or no	0.789 (0.119–3.084)	0.765
Diabetes	Yes or no	2.357 (0.332–10.8)	0.31
Hypertension	Yes or no	0.573 (0.207–1.539)	0.269
Renal insufficiency	Yes or no	2.58 (0.536–9.592)	0.183
ASO	Yes or no	4.071 (1.153–12.998)	0.021
Thrombus	Yes or no	0.421 (0.154–1.18)	0.091
Calcification	≥50% vs. <50%	3.588 (1.128–10.603)	0.023
Antiplatelet therapy	One level more	13.5 (3.689–58.315)	0
External iliac angioplasty	Yes or no	0.703 (0.254–1.886)	0.484
Crossed-limb techniques	Yes or no	1.033 (0.154–4.135)	0.967
Graft kinking	Yes or no	4.308 (1.377–13.103)	0.01
BMS	Yes or no	0.245 (0.038–0.909)	0.068

**Table 5:** Multivariate logistic regression ORs and 95% CIs for LGO, controlling for significant study covariates.

Variable	Category or increment	Multivariate OR (95%CI)	*p*-value
Sex (male)	Yes or no	5.373 (0.375–85.384)	0.204
ASO	Yes or no	0.916 (0.037–18.921)	0.954
Calcification	Yes or no	200.639 (11.3–13,124.1)	0.002
Antiplatelet therapy	One level more	25.865 (1.019–1,215.39)	0.058
Graft kinking	Yes or no	24.517 (2.718–469.522)	0.012

In total, 15 patients from the cohort were not followed up. There was a 90.4% follow-up rate. Two (1.2%) of the 13 patients (8.2%) who passed away during follow-up were in group A: one of them passed away after 40 days of all-cause care, and the other after 5 years of multiple organ dysfunction syndrome. Of the 11 (7.0%) patients from group B that passed away, 9 (5.7%) did so due to non-aortic causes, 5 died of all causes between 3 months and 2 years, 1 died of cerebral hemorrhage after 1 year, 1 died of gastric carcinoma after 2 years, 1 more patient died of nasopharynx cancer after 3 years, and 1 patient died of lung failure after 4 years. Two patients (1.2%) passed away due to aortic causes: the first patient died 2 years later from a ruptured AAA, and the second patient died 20 days following surgery from perioperative complications. During surgery, no individuals (0%) died in the BMS group, and one (1.8%) died in the non-BMS group (*p* = 1). The patient was 85 years old and had an extensive medical history, such as cerebral infarction and cardiac insufficiency. The patient suffered sudden cardiac arrest due to surgical stimulation during the operation, and the rescue was ineffective. The patient's death was not related to the operation itself.

At a mean follow-up imaging of 28.7 ± 23.6 months (range 1–124), most endograft limbs maintained patency (rates = 88.5%). The patients who received a second intervention with a BMS did not develop re-occlusion. Kaplan–Meier analysis identified patency rates at 2 years for groups A (98%) and B (84%) (*p* < 0.05) (Figure 3).

## Discussion

The LGO rate in this study was 11.5% (18/157), consistent with a range of approximately 2.7%–10.6% (3, 4). This investigation revealed that BMS application in endograft limbs could reduce the occlusion rate, consistent with the studies by Ferrer et al. (10) and Sivamurthy et al. (14). In the study by Ferrer et al., 29 individuals received high-radial force stents during the initial EVAR, all of which maintained patency. Conversely, Sivamurthy reported no occlusions in 85 limbs of patients who received adjunctive stents, compared to 13 in the unstented group (*n* = 361).

The pre-operative CT images were selected to assess the anatomical features of the IA rather than angiography (Figure 1). Oshin et al. (15) elaborated that completion angiography is a two-dimensional representation of a three-dimensional (3D) object. This angiography is inadequate for determining limbs at risk for occlusion (12). CT can perform 3D reconstruction to overcome the shortcomings of angiography.

According to the literature, the probability of limb occlusion is usually linked to anatomical and graft-related variables. More than half of the anatomical factors are vascular and include vascular calcification, iliac stenosis, small luminal diameter, and significant angulation of the iliac vessels (8, 15, 16). In this study, the univariate and multivariate analysis results showed that iliac stenosis is a risk factor for LGO. Peripheral artery disease was a risk factor for re-intervention (11). In the BMS group, the occluded patient had peripheral artery disease and CIA stenosis before the EVAR. Severe tortuosity of the iliac artery is the most critical factor responsible for LGO (15). The definition of tortuosity was different in different articles (1, 7, 12). Tortuosity in this experiment was characterized as an acute angle (more than 60°) of the CIA or when IA appeared visually doubled or more on an axial CTA slice. Graft factors encompassed stent-graft limb extension into the EIA, non-compliance with instructions for use (IFU), design, and brand of the stent (17–19). The probability of limb occlusion appears to increase as the stent-graft limb extends into the EIA. Our study found that kinking and calcification are risk factors for LGO, which is consistent with previously reported findings.

As for crossed-limb techniques, our study found no statistical difference. This is in contrast with a previous study by Yagihashi et al. (20). This may be due to the small sample size.

Among other things, a BMS is a self-expanding flexible stent that is resistant to plastic deformation. While resistance to deformation helps the stent alter the anatomy of the iliac artery, flexibility lets the stent fit the morphological characteristics of the original artery (21). A BMS creates a smooth transition between the graft limb and the native artery (15); a BMS also provides radial support to the blood vessels and reduces their elastic retraction (22). LGO also results from the internal folding of the covered stent. However, after the BMS inlay, no re-occlusions occurred (23).

The occluded patient in group A was pre-placed with a BMS and had a peripheral vascular condition. Radial force cannot be applied to cause the covered stent to collapse internally. Thus, it could be preferable to place a BMS in an IA during EVAR after the covered stent has been inflated.

In this study, the BMS group had a greater frequency of graft stents implanted in the EIA, whereas only a limited number were implanted in the non-BMS group. The LGO rate between the BMS and non-BMS groups was *p* < 0.05. Lee et al. (24) suggested that, during an EVAR of an AAA, when the covered stent extends to the EIA, an adjunctive stent should overlap within the graft and the native artery to prevent LGO. Extension of the stent-graft limb into the EIA is the independent risk factor of LGO (5). Moreover, a poor transition between the native artery and stent-graft limb may result in LGO. A BMS creates a smooth transition between the graft limb and the native artery (15). BMSs in use include Wallstent, SMART, Palmaz, and Bard stents. Sivamurthy et al. (15) chose Wallstent as an adjunctive stent, whereas our study's most commonly used stent was the Bard stent. The covered stent was not used by us for the following reasons. First, the graft stent's covered limb is the stent, the limbs typically have sufficient anchoring area, and the BMS's role is limited to radial support (22). Second, covered stents may lead to internal folding, another risk factor for LGO (23). Future research should assess the effectiveness of bare metal stents compared to coated stents in cases of severe tortuosity.

Surgical and endovascular methods are employed to manage LGO. A thrombectomy is the standard surgical procedure used to address graft thrombosis. Thrombectomy procedures were performed on a portion of the participants in this study. However, there is still a chance of limb occlusion, which might harm or interfere with the graft (25). Most patients received endovascular treatment. Those who received a second intervention with BMS did not develop re-occlusion. These treatments could reveal the cause of LGO. However, it is essential to address the risk factors to reduce re-intervention for LGO (17). When a BMS is implanted in a tortuous IA, it facilitates a seamless transition between the graft limb and the native artery, offering radial support in the stent (12). Pini et al. (26) treated LGO using a local injection of urokinase and a BMS in vascular distortion and stenosis cases.

### Limitations

There are several limitations in this study. First and foremost, it was a retrospective study; the follow-up period was lengthy and certain clinical data were lacking. Second, the study was conducted only at one location. Third, this study's sample size was limited (157 cases). Fourth, the BMSs used were from a single company and without the comparison with a covered stent.

## Conclusion

To prevent limb occlusion, it is safe and successful to deploy a BMS into severely tortuosity iliac arteries. This method has apparent long-term effects in terms of patency and re-interventions.

## Data Availability

The original contributions presented in the study are included in the article/Supplementary Material, further inquiries can be directed to the corresponding author.
